# Data on galvanic-evoked head movements in healthy and unilaterally labyrinthectomized rats

**DOI:** 10.1016/j.dib.2016.08.048

**Published:** 2016-08-31

**Authors:** Moslem Shaabani, Yones Lotfi, Seyed Morteza Karimian, Mehdi Rahgozar, Mehdi Hooshmandi

**Affiliations:** a Audiology department, University of Social Welfare and Rehabilitation Sciences, Tehran, Iran; b Department of Physiology, School of Medicine, Tehran University of Medical Sciences, Tehran, Iran; c Biostatistics Department, University of Social Welfare and Rehabilitation Sciences, Tehran, Iran; d Department of Physiology, Medical School, Shahid Beheshti University of Medical Sciences, Tehran, Iran

**Keywords:** Galvanic vestibular stimulation, Galvanic-evoked head movement, Vestibule, Rat

## Abstract

In this dataset, we analyzed galvanic-evoked head movements (GEHMs) in the spatial planes of yaw, and roll in normal and unilaterally labyrinthectomized (UL) Wistar rats. The rats were assigned in 4 groups of 10: control, sham, right-UL and left-UL. Bilateral galvanic vestibular stimulation (GVS) was presented by our “ring-shaped electrode” design (see “*Short-term galvanic vestibular stimulation promotes functional recovery and neurogenesis in unilaterally labyrinthectomized rats*” (M. Shaabani et al., 2016) [1]). Required data were collected through video recording of GEHMs followed by image processing and statistical analysis.

**Specifications Table**

**Table:** 

Subject area	*Biology*
More specific subject area	*Vestibular system*
Type of data	*Graph, figure*
How data was acquired	*Survey*
Data format	*Analyzed*
Experimental factors	*Unilateral chemical labyrinthectomy and subcutaneously implanting galvanic stimulating electrodes in rats*
Experimental features	*Analysis of the degree and plane of galvanic-evoked head movements in the threshold and suprathreshold levels via image processing*
Data source location	*Tehran, Iran*
Data accessibility	*Data is with this article*

**Value of the data**

• Ring-shaped electrodes provide the opportunity of stimulating unrestrained rats.
• The plane of galvanic-evoked head movement (GEHM) could be utilized as a marker of the activated vestibular afferents in normal and unilaterally labyrinthectomized (UL) rats.
• GEHM could be applied as a confirmation test for an induced chemical UL in rats.
• Contralesional GEHM in the UL rats could be used as a therapeutic strategy.

## 1 Data

The data refers to the degree and spatial planes of head movements provoked by GVS at the threshold level (GEHM-threshold) and suprathreshold level (suprathreshold-GEHM) in healthy and arsanilate-induced UL rats.

## 2 Experimental design, materials and methods

Forty male Wistar rats (180–220 g, Pasteur Institute of Iran) were used. Twenty rats were randomly modeled as UL: 10 rats modeled as right-UL (RL group), and 10 rats as left-UL (LL group). The other twenty rats were randomly assigned to control group (intact) and sham group.

Animals were housed 3–4 per cage under constant temperature (22±1 °C) with 12 h light /dark cycle. Food and water were freely accessible.

### 2.1 Chemical labyrinthectomy

Unilateral labyrinthectomy (UL) was carried out by intratympanic injection of sodium arsanilate following the approach we reported in [1]. Briefly, rats (UL and sham groups) were intraperitoneally anesthetized by a solution of Ketamine (100 mg/kg, Alfasan, Netherlands) and Xylazine (10 mg/kg, Alfasan, Netherlands). For preparing arsanilate solution, 300 mg of sodium arsanilate (Sigma-Aldrich) dissolved in 1 ml of 0.9% saline solution. Each UL rat was transtympanically (TT) received a single-dose (0.1 ml) of the arsanilate solution in the ipsilesional tympanic cavity (i.e. the right-ear in RL group and the left-ear in LL group) while underwent a 30–45 min anesthesia. Five rats of sham group received a single-dose (0.1 ml) of saline solution (0.9%) in the right-tympanic cavity and 5 of them received that in their left-ear.

### 2.2 Behavioral evaluation for confirmation of UL

In addition to the behavioral observation to inspect head-tilt and rotational locomotion toward the UL side [2], [3], [4], the landing and air-righting reflexes were examined for confirmation of UL. The details of these examinations were reported in [1]. The score of “2” in both tests was considered as “abnormal” and confirmed the UL.

### 2.3 Electrode preparation and implantation

**Fig. 1:**
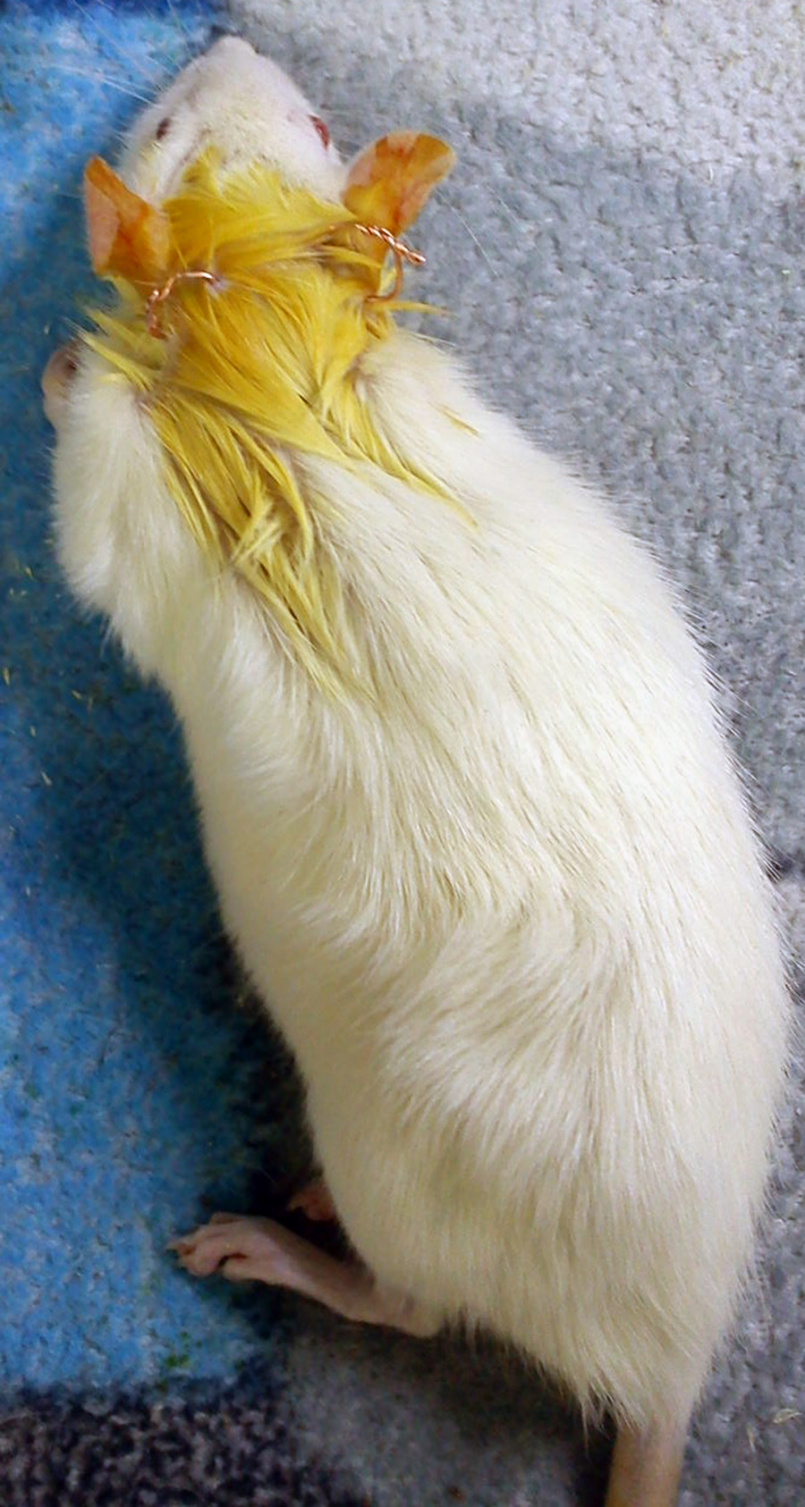
**Ring electrodes.** The copper-made electrodes were subcutaneously inserted behind each ear. This electrode type enabled us for stimulating the freely-moving rats.

The method of electrode implantation was thoroughly described in [1]. Briefly, short lengths of an uninsulated copper wire (with diameter of about 1 mm) were subcutaneously inserted behind each ear, by using an angiocath needle. Then, through twisting the free tails of the wire, the "ring-shaped electrode" was created and the alligator-connectors of our GVS device was attached to it. The length of subcutaneous part was about 5 mm (Fig. 1).

### 2.4 GEHM-threshold

**Fig. 2:**
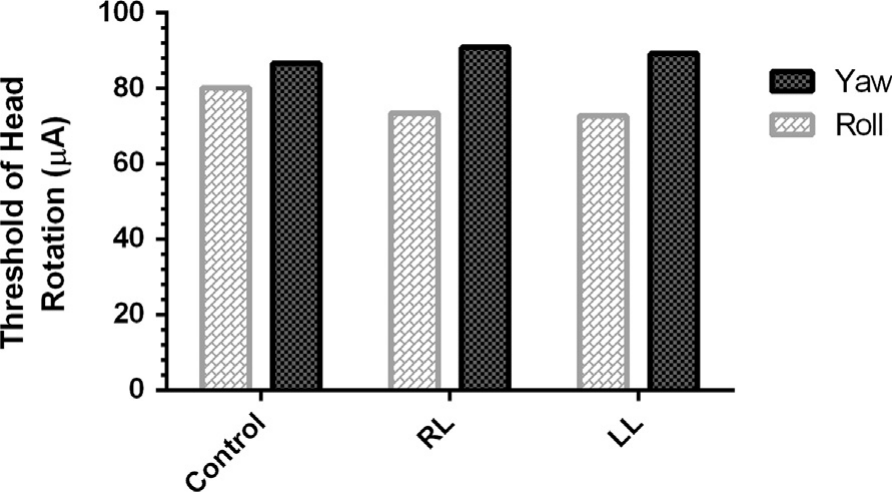
**GEHM-threshold.** This graph shows the threshold of GEHM for spatial planes of yaw and roll in the control, RL, and LL groups. LL: Left labyrinthectomized; RL: Right labyrinthectomized.

Bilateral GVS (rectangular pulses, 1–2 Hz random frequency, and 5-ms pulse duration) was delivered for detecting thresholds in alert rats which performed just a week after TT injection. Randomized frequency was selected for preventing any potential effect of adaptation. The left and the right mastoids were selected for anodal stimulation in the RL and LL groups, respectively. The same protocol was used for the sham group. In the control group, half was tested with the left mastoid as anodal stimulation and the other half with the right mastoid as anodal stimulation. The GVS-intensity was gradually increased from zero until an obvious repeatable 1–2 Hz galvanic evoked head movement (GEHM) was clearly observed. The lowest intensity that induced an apparent GEHM considered as the GVS-threshold. It should be noted that the GVS-intensity was increased too enough until detection of GEHM-threshold in the two spatial planes for each rat (Fig. 2). The detail of recording technique is described under the title of "Recording procedure of GEHM".

### 2.5 Suprathreshold-GEHM

**Fig. 3:**
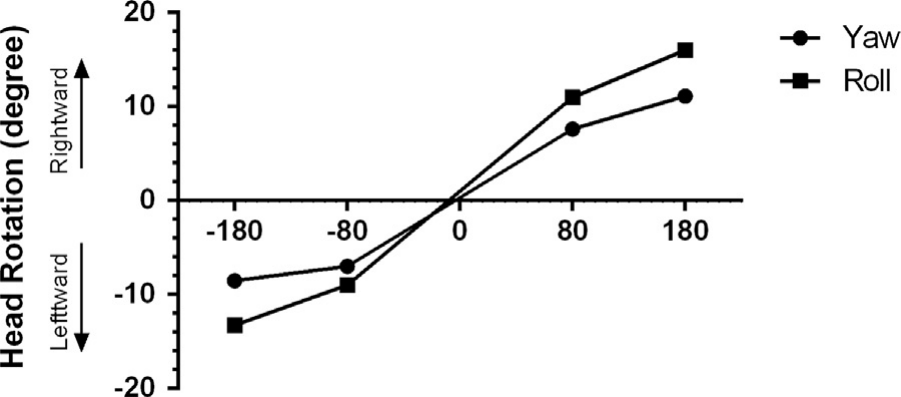
**Suprathreshold-GEHM evaluation in the control group.** Each trace shows the mean angular head rotation within each cardinal plane (yaw and roll) as a function of current intensity and polarity.

**Fig. 4:**
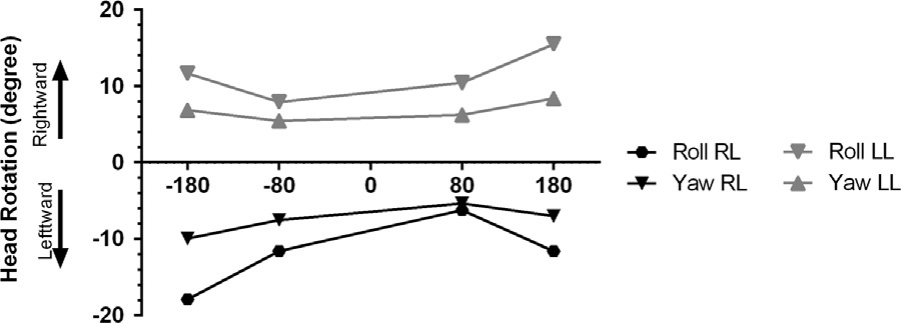
**Suprathreshold-GEHM evaluation in the UL groups.** Each trace shows the mean angular head rotation within each cardinal plane (yaw and roll) as a function of current intensity and polarity in the LL and RL groups. LL: Left labyrinthectomized; RL: Right labyrinthectomized.

This evaluation also performed just a week after TT injection. For this part of study on the alert rats, bilateral GVS (rectangular pulses, 1–2 Hz random frequency, and 5-ms pulse duration) was presented for 40 min. In fact, the total time was divided into four 10-minute epochs that randomly delivered to each rat: a. 80 μA and right-ear as anode; b. 180 μA and right-ear as anode; c. 80 μA and left-ear as anode; and d. 180 μA and left-ear as anode (Fig. 3; Fig. 4). Between every two epochs, a rest time of 5 min was prearranged. Randomized frequency, random delivery of 4 epochs, and rest time were considered for preventing any potential effect of adaptation and habituation. As mentioned above, the detail of recording technique is described below.

### 2.6 Recording procedure of GEHM

**Fig. 5:**
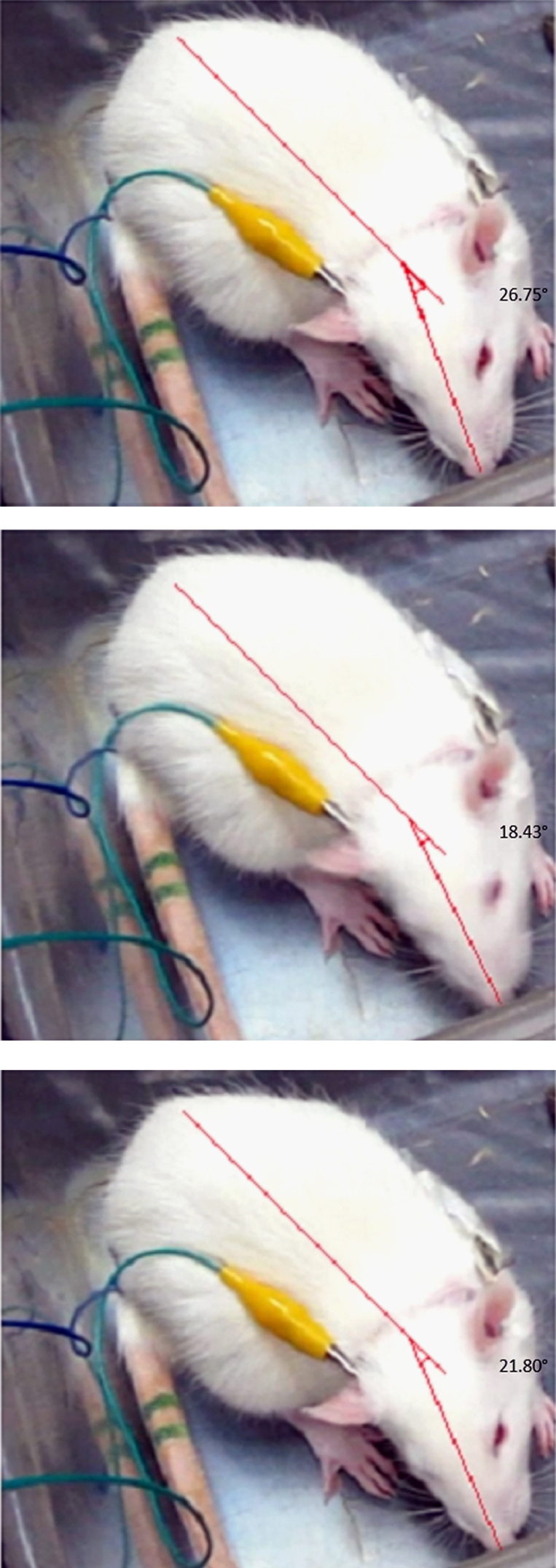
**Measurement of GEHM degree in the roll plane.** Each GEHM event divided into consecutive sequences by means of Corel VideoStudio software. Then, the degree of head rotation was measured by fitting of required reference lines on the snout and body of rats via Dinolite Digital Microscope software. The image series in this figure were selected from a GEHM evaluation on a right labyrinthectomized rat.

**Fig. 6:**
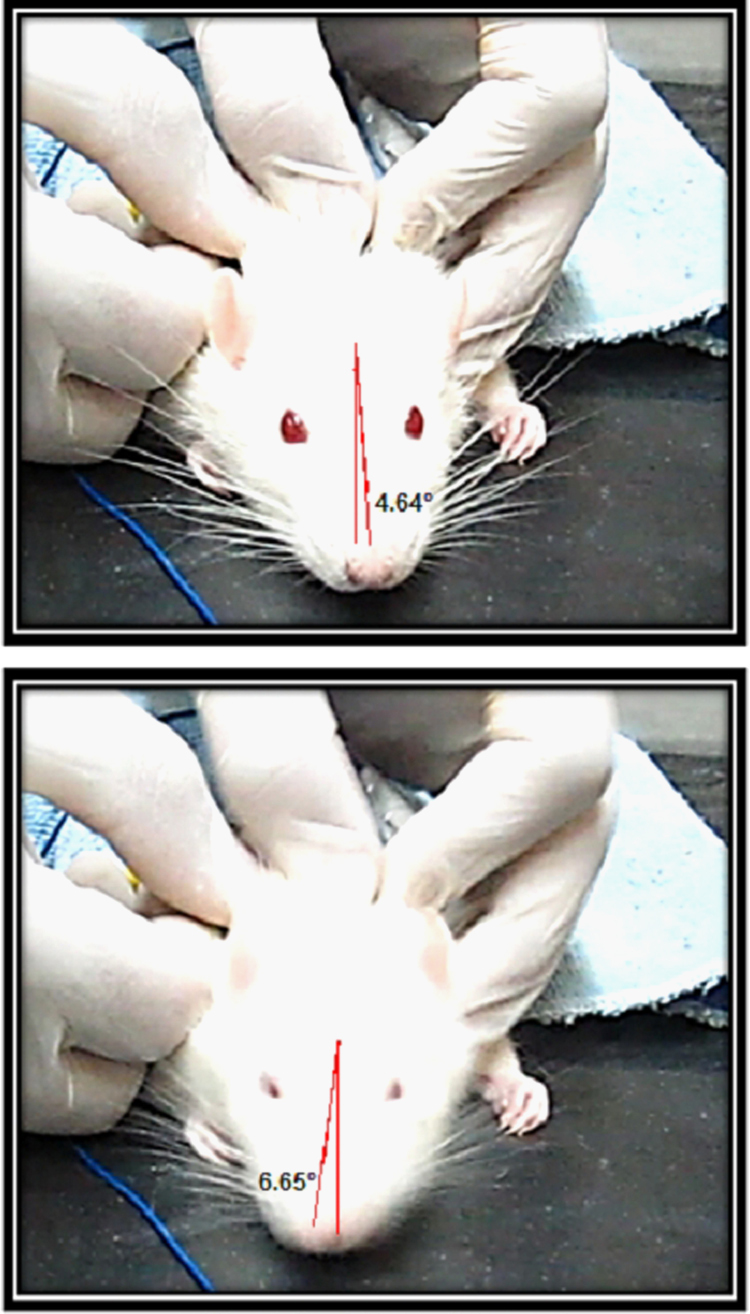
**Measurement of GEHM degree in the yaw plane.** These two consecutive pictures were selected from a GEHM evaluation on a control rat.

All investigations, including evaluation of GEHM-threshold and Suprathreshold-GEHM, were recorded by a video recorder camera (Canon, IXUS 100 IS, 12.1 megapixels, 30 fps) focused on the rat’s head. For each rat, 5 GEHM in each epoch were selected by slowing down video clips, cutting and extracting the pictures using Corel VideoStudio software (ver. Pro X5). Then, the degree and plane of head rotation in each selected pictures were precisely determined by the fitting of measurement tools (such as reference lines and angles) on the head, snout, ears and body of rats via Dinolite Digital Microscope software (ver. 3.2.0.5) (Fig. 5; Fig. 6). Afterwards the calculated degree of all 5 GEHM in every epoch was averaged to estimate a mean-GEHM in each cardinal plane for each rat.

### 2.7 Statistical analysis

For comparing the mean GEHM-threshold (in the 2 cardinal planes among the 4 groups) and the mean degree of suprathreshold-GEHM (in the 2 cardinal planes and 4 stimulus conditions among the 4 groups), repeated measures ANOVA were used. GraphPad Prism 6 (GraphPad Software, Inc., CA, USA) was used for the analysis and preparing graphs.
